# A Complex Dance: Measuring the Multidimensional Worlds of Influenza Virus Evolution and Anti-Influenza Immune Responses

**DOI:** 10.3390/pathogens8040238

**Published:** 2019-11-15

**Authors:** Jiong Wang, Alexander Wiltse, Martin S. Zand

**Affiliations:** 1 Department of Medicine, Division of Nephrology, University of Rochester Medical Center, Rochester, NY 14534, USA; Alexander_Wiltse@urmc.rochester.edu (A.W.); Martin_zand@urmc.rochester.edu (M.S.Z.); 2 Clinical and Translational Science Institute, University of Rochester Medical Center, Rochester, NY 14534, USA

**Keywords:** influenza virus, humoral response, hemagglutinin (HA) of influenza virus, broad neutralizing antibody(bnAb), heterosubtypic immunity of influenza, original antigenic sin “OAS”, “universal” influenza vaccine, protein microarray assay, mPLEX-Flu assay, multiple dimensional assay (MDA)

## Abstract

The human antibody response to influenza virus infection or vaccination is as complicated as it is essential for protection against flu. The constant antigenic changes of the virus to escape human herd immunity hinder the yearly selection of vaccine strains since it is hard to predict which virus strains will circulate for the coming flu season. A “universal” influenza vaccine that could induce broad cross-influenza subtype protection would help to address this issue. However, the human antibody response is intricate and often obscure, with factors such as antigenic seniority or original antigenic sin (OAS), and back-boosting ensuring that each person mounts a unique immune response to infection or vaccination with any new influenza virus strain. Notably, the effects of existing antibodies on cross-protective immunity after repeated vaccinations are unclear. More research is needed to characterize the mechanisms at play, but traditional assays such as hemagglutinin inhibition (HAI) and microneutralization (MN) are excessively limited in scope and too resource-intensive to effectively meet this challenge. In the past ten years, new multiple dimensional assays (MDAs) have been developed to help overcome these problems by simultaneously measuring antibodies against a large panel of influenza hemagglutinin (HA) proteins with a minimal amount of sample in a high throughput way. MDAs will likely be a powerful tool for accelerating the study of the humoral immune response to influenza vaccination and the development of a universal influenza vaccine.

## 1. Introduction

Influenza is a global public health problem, causing approximately 300,000–650,000 global deaths each year [1]. Influenza A and B are the major virus types that infect humans. Antibodies directed against the head domain of the surface glycoprotein hemagglutinin (HA) of influenza virus have proven to be the major source of protective immunity, blocking viral binding to the receptors on the target human cell surface and inhibiting viral entry to target cells. In response to human immunity pressures, antigenically distinct influenza viruses emerge frequently, caused by continual mutation (antigenic drift) [2], or reassortment among viruses from different species (antigenic shift) that can lead to a pandemic with high mortality [3,4].

To date, seasonal influenza vaccines composed of three or four inactivated virus strains are the only licensed vaccines to elicit or boost protective immunity against influenza viruses in the United States. However, both antigenic drift and shift necessitate that the flu vaccine be reformulated and re-administered annually [5]. It is a formidable challenge to select the strains each year to protect against current circulating viruses based on viral surveillance data of the previous year [6], and to produce a large amount of antigenically matched vaccine. Developing a “universal flu vaccine” that induces broadly cross-protective immunity is one strategy to overcome this challenge [7,8].

Antibody mediated immune responses against influenza HA are multi-dimensional, targeting multiple antigenic determinants (epitopes) within the HA molecule. Antibody mediated responses are also incredibly complicated, as they are influenced and altered by an individual’s prior influenza exposure history. This includes factors such as “original antigenic sin” (OAS) [9] (also known as HA imprinting [10]) and the shared epitopes between proteins from different influenza strains that induce cross-strain immunity, such as heterosubtypic immunity [11,12]. The effects of pre-existing antibodies on the B cell response to vaccine strains that contain HA antigenic sites similar to those from prior exposures are still unclear. Systems serology, the application of bioinformatics to multidimensional data regarding anti-influenza IgG binding specificity and repertoire in response to vaccination, has emerged as a way to understand these responses, and to aid in vaccine design.

Because of the complex interplay between pre-existing, circulating, anti-HA antibodies and human IgG-mediated influenza responses, the first step in comprehensive analysis is measurement of anti-influenza HA IgG binding patterns against multiple influenza strain HAs. Such measurement is referred to as multi-dimensional, referring to the multiplicity of influenza strain binding reactions quantified. Such measurements are critical for understanding how IgG recognition of shared epitopes across influenza strains can lead to cross-strain protection, and for better defining the functional host anti-HA influenza repertoire.

Various assays exist to measure the host anti-HA influenza antibody response. The assays currently used to estimate the HA IgG antibody binding to single HA proteins, such as hemagglutinin inhibition (HAI) [13,14], micro-neutralization (MN) [15,16] and enzyme-linked immunosorbent assay (ELISA), all require a large amount of serum sample in order to test the cross-reactivity against an array of virus strains. These assays are also expensive and time consuming, limiting their usefulness in unraveling the complexity of cross-reactive antibody patterns to influenza viruses. In contrast, the novel technology of array-based high throughput multiple dimensional assay (MDA) provides a powerful tool to comprehensively analyze the presence and effects of broad cross-reactive antibodies (bcAbs) against the influenza HA protein.

Here, we review the genetic foundations of shared epitopes leading to IgG cross-reactivity between antigenically similar influenza virus strains, and the contributions of these cross-reactivities to OAS and subsequent host immune responses to influenza infection and vaccination. The remainder of the review discusses the technology of MDA, and highlights the application of MDA as a powerful tool for future influenza immunity studies and universal vaccine development.

## 2. Hemagglutinin (HA) and Its Antibodies

Influenza viruses, the pathogens that cause flu, belong to the *Orthomyxoviridae* family, a group of negative-sense single strain RNA viruses [17]. Influenza type A has two phylogenetic groups based on amino acid sequence and, to date, 18 HA subtypes: Group 1 (H1, H2, H5, H6, H8, H9, H11, H12, H13, H16, HA-like H17, and HA-like H18) and Group 2 (H3, H4, H7, H10, H14, and H15) [18]. Influenza A viruses are further named based on the composition of major surface glycoproteins HA and neuraminidase (NA) (e.g., H1N1 or H3N2). Influenza type B also has two phylogenetically distinct lineages called Yamagata and Victoria [19]. The major source of human protective immunity is the antibodies directed against the head domain of the HA of influenza virus [20]. HA is the most abundant influenza viral surface glycoprotein and mediates binding to sialic acid expressed on the surface of target host cells. HA is synthesized as a polypeptide (HA0) before being cleaved into HA1 and HA2 subunits, which fold into a trimeric spike. The membrane distal globular head region of HA is composed of HA1 and contains the receptor binding site (RBS) that the virus uses to bind to host cell sialic acid. The stalk region then mediates virus fusion into host cells through structure transformation [21].

Protective antibody-mediated immunity against HA is the first line of defense in preventing influenza virus infection. Such immunity is elicited by prior influenza exposure: infection or vaccination [22,23]. Anti-head HA antibodies typically target epitopes in and around the RBS. Five major B cell epitopes have been identified for H1 (Sa, Sb, Ca1, Ca2, and Cb) [24] and H3 (Eptitopes A–E) influenza strain HAs [25]. The HA head region, formed by HA1, is strongly immunodominant, highly mutable, and strain-specific [26]. The HA stalk region, formed by HA2 as well as the N- and C-terminal ends of HA1 in an alpha-helical structure, supports the head region of HA [27]. The highly conserved nature of the HA stalk makes it a promising target for universal influenza vaccines [28,29,30].

The goal of universal vaccines is to elicit protective broad cross-reactive antibodies (bcAbs), especially broad neutralizing antibodies (bnAbs). Most head-reactive antibodies are not bcAbs or bnAbs but rather strain-specific. However, more and more head domain recognizing bnAbs have been identified, such as KBm2, 5J8 and CH65, which neutralize a broad spectrum of H1 strain viruses in the MN assay [31,32,33], and 8M2, which neutralizes many H2 strains [34]. Several head-reactive bnAbs demonstrate heterosubtypic reactivity, such as C05, F045-92 and S139/1, which recognize the conserved receptor binding pocket on the HA head [35,36,37,38]. These three bnAbs can neutralize H1, H2, and H9, while C05 can also weakly neutralize the Group 2 H3 influenza virus [35].

Recently, an increasing number of bnAbs have been isolated and identified from the B cell repertoire after influenza virus infection and vaccination [31,39], targeting both head and stalk regions of HA. Some bnAbs that target stalk region of HA neutralize a wider range of influenza types and subtypes [18]. Human monoclonal antibodies CR6261, F10 and A06 were isolated from recently vaccinated donors and shown to neutralize nearly all Group 1 viruses [40,41,42], while CR8020 and CR8043 neutralize a wide breadth of Group 2 viruses [43,44]. Some monoclonal antibodies show broad cross-group influenza A reactivity, including MEDI8852, 27F3, FI6v3 and CR9114 [45,46,47,48]. Notably, there are other in vivo mechanisms involved in antibody mediated broad-protection, such antibody-dependent cell-mediated cytotoxicity (ADCC) and antibody-mediated cellular phagocytosis (ADCP) [49,50,51]. For example, the novel isolated human mAb FluA-20 was shown to protect mice against lethal challenge with H1, H3, H5 and H7 influenza A subtype viruses [51]. It binds to an extremely well conserved epitope in the peripheral interface of the HA trimer, a novel epitope on the head region of HA, with extra high affinity. After binding to HA, it quickly interferes with the trimeric structure of HA, which blocks viral cell-to-cell spread. This mAb offered protection from influenza virus infection in vivo, but it did not display neutralizing activity in HAI or MN assay in vitro studies [51]. This suggests that there are some bcAbs that are able to protect against influenza virus that would likely be disregarded by traditional assays. BcAbs can be detected by ELISA and other binding assays, including MDAs, thus highlighting the need for more sensitive assays in the search for broad cross-reactive antibodies.

One major issue with the evaluation of bnAb activities by traditional HAI and MN assays is that they can only measure the magnitude of bnAb against specific virus strains, and cannot determine the breadth of bnAbs against a large panel of influenza strains. In addition, during antibody screening, these assays most likely omit bcAbs, such as the human mAb FluA-20 [51], as we discussed above, did not display neutralizing activity in MN assay in vitro.

## 3. Complexity of Human Immune Responses against Influenza Virus

The complexity of the human immune response to repeated influenza virus exposure is another major obstacle to the development of a universal vaccine. Because of the frequent antigenic drift in circulating influenza strains, humans have more complicated immune responses than can be modeled in naive animals. Each person has a unique history of influenza virus exposure, leading to pre-existing immune repertoires that are activated in the event of an immune challenge with an antigenically similar flu strain.

In 1960, Thomas Francis Jr. reported that antibodies against the first H1N1 flu strain encountered in life would be produced at high levels throughout a person’s lifespan, to the detriment of future specific responses to new strains [9]. He coined the term “Original Antigenic Sin” (OAS), now referred to as imprinting, to describe how a specific immune response to a flu strain can be preferentially directed at a previously encountered strain. This phenomenon also relates to the cross-protection provided by pre-existing bcAbs and how the breadth and protective potency of cross-reactive immunity is enhanced by infection or vaccinations. For example, the lower mortality of older individuals during the 2009 H1N1 pandemic is attributed to the structural similarity between the pandemic 2009 “Swine” flu virus HA and the pandemic 1918 “Spanish” flu virus HA, suggesting within-subtype cross-strain protection [52,53,54]. Importantly, in 2016, using all known fatal human cases of H5N1 and H7N9, Gostic et al. [10] found that childhood H1 and H3 imprinting provided 75% and 80% protection against death from H5N1 and H7N9, respectively. Because H1 and H5 are found in phylogenetic Group 1, and H3 and H7 are found in Group 2, these results suggest that antigenic seniority boosts can offer cross-protection against HA subtypes of the same group [10]. The mechanism of such immune imprinting is unclear, but it has been hypothesized that after a large number of memory B cells (MBCs) are activated during first influenza virus exposure, the next exposure to an influenza strain with some mutated and some shared epitopes will show lower de novo naive memory B cells activation against the *new* epitopes. This suggests that preexisting antibodies could play a role in the MBC response, such as accelerating the clearance of influenza antigens or sterically blocking MBCs from accessing specific epitopes [55,56]. Meanwhile, the MBCs specific for epitopes present in the first strain would proliferate more since they have been activated again [57]. OAS [58], “antigenic seniority” [59] and HA imprinting [10] try to describe the effects of pre-existing antibodies on the antibody response to similar or related influenza virus strains. Due to the multi-dimensional nature of human immune repertoires, single-dimensional assays are extremely limited in their ability to measure the breadth of pre-existing bcAb and MBC responses. MDAs, on the other hand, are an ideal tool for measuring pre-existing bcAb profiles and broad influenza immunity.

## 4. Multidimensional Assays (MDAs) for Anti-Influenza Antibodies

The gold standard and most widely used assays to evaluate antibody activity against HA and protection in clinical trials are HAI [13,14] and MN [15,16] assays. Both assays are semi-quantitative with a single target virus strain providing a discrete ranked readout of one of 8–14 titer values based on two-fold dilutions of serum samples. Including ELISA, which is less frequently used in influenza studies, all these common methods are single dimensional assays, which require the user to perform antibody testing for each strain of interest separately. This process is not only time-consuming and labor-intensive, but also requires large sample volumes. In addition, these assays are limited in their ability to show the breadth of cross-reactive anti-influenza antibody response.

To overcome the limitations of single dimensional assays, novel multidimensional assays (MDAs) have been developed over the last decade. MDAs are high throughput assays that use protein array technology to simultaneously measure antibodies against a panel of the HA proteins and peptides of multiple influenza virus strains in a single test with minimum amount of sample. They can measure the magnitude and breadth of antibody response against HAs of influenza virus. In general, the purified HA proteins are immobilized on a solid surface such as microchips, membranes or beads, to keep the native structure and provide their maximal binding properties. Then, the reactive antibodies are characterized by binding to the protein, followed by a fluorescent probed secondary antibody that is read by an array chips reader as median fluorescent intensity (MFI). The HA protein or peptide array offers the advantage of multiplex capabilities to generate statistically powerful data while conserving time, money, and requiring minimal sample compared to the traditional assays. While not a functional assay, multiple studies have confirmed that MDA results correlate well with HAI titers [60,61,62,63,64]. Critical for the understanding of OAS, “back-boosting”, and the effects of pre-existing cross-strain immunity on current vaccine responses, such methods allow testing reactivity against a large number of antigenically related and disparate influenza proteins (generally HA at the moment).

Based on the immobilizing materials, there are two major types of array-based assays currently used for evaluation of HA antibodies: protein microarray and Luminex assays. The first method involves printing HA protein on chips to estimate the binding antibodies. The first report of HA protein array assay was published in 2010 [65], and since then more than 10 publications have shown its powerful potential to study the breadth of cross-reactivity of HA antibodies on the population level (see the list in Table 1). At present, 283 HA proteins can be printed on one microchip for maximum efficiency [66]. However, this process requires expensive and specialized equipment, including a micro-printer and dedicated scanner.

In contrast to peptide arrays, the Luminex-based MDA method, which involves coupling HA protein to color coded Luminex beads, allows the user increased flexibility to customize the panel by easily combining multiple strain-specific beads without reprinting the entire panel [63,67]. In addition, Luminex readers are more widely available now than chip scanners. However, Luminex-based MDAs support fewer analytes per assay. For example, the Luminex 200 can detect 100 color-coded beads, and the Magpix analyzer can read 50 coded beads [68].

**Figure 1:**
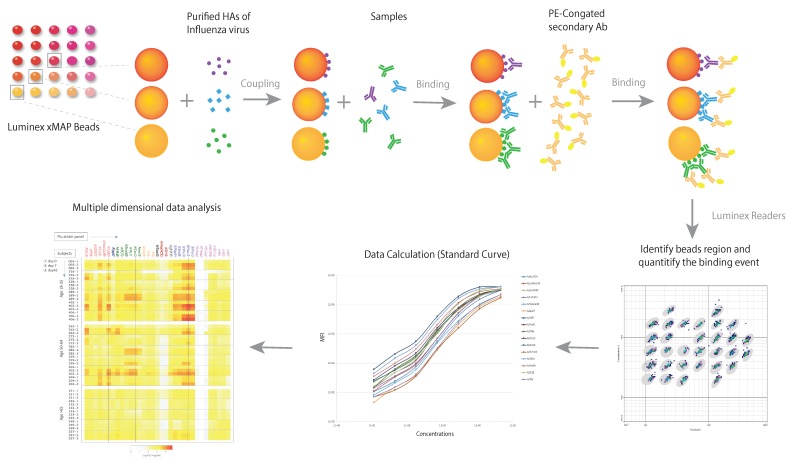
The principle of a multiple dimensional assay (MDA). The schematic diagram demonstrates the principle of the mPLEX-Flu assay, an example of a MDA, which is a Luminex based assay. Each color-coded xMAP bead is coupled with the purified recombinant hemagglutinin (rHAs) of one influenza virus strain. The different colors of influenza strain HA-specific beads are mixed and incubated with the serum sample. Bound anti-HA antibodies are subsequently detected using detection antibodies specific to each antibody isotope. The magnetic beads are read on a dual-laser flow-based Luminex reader. One laser classifies the bead and identifies the analyte being detected. The second laser determines the magnitude of the PE-derived signal, which is in direct proportion to the amount of analyte bound. Median fluorescence intensity (MFI) is converted to absolute concentration using a standard curve, at which point multiple dimensional data analysis can begin.

The first Luminex-based MDA, mPLEX-Flu, was developed to characterize the breadth and magnitude of the IgA, IgM and IgG antibodies against a large panel of whole HA proteins of multiple influenza virus types and subtypes in 2015 [67]. Our recent comprehensive studies, with novel statistical methods and a continuous readout across a 4.5 log range, indicated that MDA highly correlated with HAI and MN results, and with substantially better sensitivity and precision on account of continuous readout [64]. Furthermore, another study showed that using individual standard curves for each influenza HA strain in the mPlex-Flu assay to independently calculate IgG concentrations against each virus strain enables the direct comparison of serum anti-HA IgG concentrations against different influenza HA subtypes [76]. This ability addresses an essential issue for estimation and comparison of cross-reactivities of influenza antibody against multiple strains that has always plagued single-dimensional assays including HAI, MN and ELISA. The principle of the mPLEX-Flu assay is shown in Figure 1.

**Figure 2:**
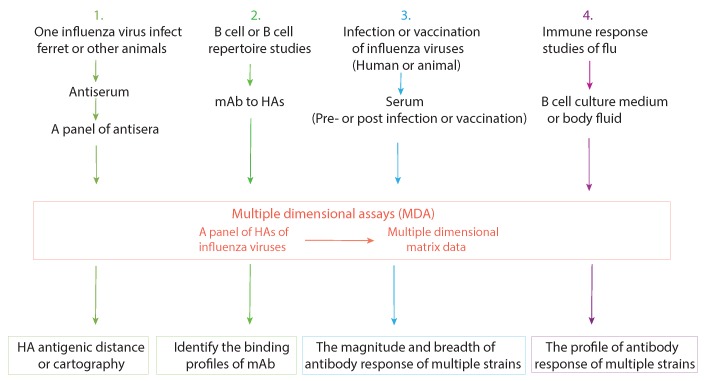
The current applications of multiple dimensional assays (MDAs). Four major influenza research applications of MDAs are listed, along with the multidimensional data set that each can generate.

Based on the above characteristics of MDA, we use the example of a Luminex-based MDA, the mPLEX-Flu assay [67], to discuss the application of MDA on influenza vaccine development and basic viral immunity research. The major applications are summarized in Figure 2.

## 5. Current Applications of MDA

### 5.1. Determination of the Antigenicity of HA of Influenza Virus

Antigenic cartography was first presented by Smith et al. in 2004 [88] as a way to quantify and visualize the antigenic differences in evolving flu strains. They used antigenic data from 35 years of H3 influenza surveillance, which consisted of hemagglutination inhibition (HI) titers multidimensional matrix data from 79 ferret polyclonal antisera against a panel of 273 viral isolates. They used multidimensional scaling analysis to adjust the position of viruses on an antigenic map such that the linear distance between two points reflects antigenic difference (calculated by comparing HI titers against other virus strains). The map revealed the high-level antigenic evolution of H3 influenza viruses from 1968 to 2002. To increase the efficiency and power of antigenic cartography, MDA could be used instead of HI to generate the multiple dimensional matrix data that could reveal the antigenic distance of HAs between the variants of influenza viruses, a technique we demonstrated in 2015 [67,71]. In the study of antigenic drift in 2015–2016 seasonal H1N1 viruses from the pandemic 2009 H1N1 virus [73], the mPLEX-Flu assay was sensitive enough to detect the antigenic difference of circulation isolates with H1 vaccine strain.

### 5.2. Identify the Binding Profiles of Broad Cross-Reactive mAb

Isolation and analysis of human monoclonal antibodies from the B cell repertoire of infected or vaccinated individuals is an important method to measure the B cell response against influenza virus [26,39,89,90]. As discussed above, broad cross-reactive antibodies (bcAbs) that are able to protect against influenza virus infection include the broad neutralizing antibodies (bnAbs) and other cross-reactive antibodies. bcAbs can be estimated by ELISA and MDA binding assays. Based on breadth of binding, bcAbs can be grouped as homosubtypic (cross-strain reactive within the same subtype groups), heterosubtypic (cross-reactive between subtypes), and heterophylogenic (cross-reactive across the phylogenic groups). MDAs can generate comprehensive high throughput data to determine the broad binding profile for mAb with tremendous efficiency. That will help accelerate research in this field. For example, KPF1, a human monoclonal Ab, was isolated by Kobies’s lab from a subject who was immunized with the seasonal influenza inactivated vaccine [31]. Using the mPlex-Flu assay, the broad binding profile of KPF1 was efficiently clarified. Multiple dimensional data characterizing KPF1 clearly showed the magnitude and breadth of cross-reactivity of KPF1, and permitted visualization of results by a heat-map graph [31]. Importantly, the mplex-Flu assay revealed that the distinct binding profile of each mAb was different from the others, even though they were isolated from same influenza infection. This type of experiment demonstrates the utility of MDA assays in rapidly defining the immune repertoire landscape against multi-antigen HA proteins of multiple influenza strains.

### 5.3. Detection of the Magnitude and Breadth of Serologic Responses to Influenza Infection or Vaccination

The major goal in developing the mPlex-Flu assay is to quantitatively evaluate the cross-reactivity of influenza virus antibodies, including IgG, IgA and IgM isotypes. After we established and verified the mPlex-Flu assay, we applied it for detection of a breadth of cross-reactive Abs elicited by infection of influenza virus or vaccination with recombinant HA proteins [63] in mice and ferrets. The assay also provides a comprehensive and efficient way to evaluate the change of broad cross-reactive humoral immunity after influenza virus infection or vaccination in human clinical studies [72,91]. One of the most extensive benefits of the application of mPlex-Flu assay to studies of the antibody response of influenza is to provide more comprehensive data for baseline, before vaccination or infection [64,76]. The high throughput data of antibody titers helps to improve our understanding of the effects of influenza virus exposure history, or OAS that we discuss above, as essential factors that shape an individual’s response to influenza vaccines or infections.

### 5.4. Detection of Antibodies in B Cell Culture Medium and Body Fluid

The high sensitivity and minimal sample size requirement enable MDA to quantitatively detect multiple influenza virus antibodies in samples other than serum (i.e., B-cell culture medium [72,74] and breast milk [75]), which contain low antibody concentration, and with small amount of sample volume, limit of detection for HAI, and MN single-panel traditional assays. Development of MBCs and activation of preexisting MBCs are essential features of the B cell response to influenza virus infection and vaccination [90,92]. Analyzing Abs in the culture supernatants of stimulated MBCs provides an alternative to ELISpot assay as a readout for HA-specific MBC responses, and facilitates a more comprehensive analysis of MBC repertoire [72]. HA-specific IgG concentrations in B cell culture medium are highly correlated with the frequencies of antigen-specific IgG secreting B cells derived from stimulated MBCs or plasmablasts [63,72,93].

As an example of the utility of combining MDA and in vitro culture experiments, we have previously analyzed low volume B cell culture samples using the mPLEX-Flu assay for changes in the size and character of HA-reactive MBC populations after H3N2 influenza infection [72] and seasonal flu vaccination [74] in a far more efficient and extensive way than could be accomplished with HAI or MN. We found that the H3-reactive IgG MBC population was expanded after infection induced reactivity to HA head and stalk domains, and head-reactive MBC populations were broad and reflected prior imprinting patterns of IgG production, which suggested that early-life H3N2 exposure affected H3 stalk-specific MBC expansion [72,74]. Similarly, a study examining the correlation between maternal and infant serum and maternal breast milk anti-influenza HA IgG and IgA patterns during the first 12 months of life showed that breast milk influenza HA-specific IgG and IgA antibody levels and patterns in breast milk were correlated with those in serum, except some H5, H4 and H9 HA head-specific Abs. A steady decay of infant influenza specific IgG levels by 6–8 months of age was also detected. This study strongly suggested that this new method could be used in a larger clinical study to understand the impact of maternal imprinting and temporary passive immunity on influenza immunity in the future [75].

## 6. Future Applications of MDA

### 6.1. Population Studies with Micro-Sampling Techniques

When coupled with low volume sampling methods, MDA has the potential to vastly increase subject sampling numbers for population based studies, while simultaneously yielding comprehensive data regarding IgG reactivity against multiple influenza strains. For example, a 2014 study used a protein microarray to monitor the trends of the 2009 influenza A (H1N1) pdm virus in 13 countries from five continents by screening bloodspots [78]. Similarly, a new technique called volumetric absorptive microsampling (VAMS), which provides for accurate sampling of a fixed blood volume (10 or 20 μL) on a volumetric swab and allows for long-term sample storage, has been used for peripheral blood sampling [94]. Combining this method with the mPlex-Flu assay enabled us to measure multidimensional anti-influenza IgG activity in whole blood samples collected by a finger-stick [95]. This study indicated that results from testing VAMS and traditional serum samples were highly correlated, both within subjects and across all influenza strains [95]. In addition, after adjustment for the hematocrit effects on the serum volume of whole blood samples, this new method accurately estimated the HA-specific IgG absolute concentration equivalent to that obtained with serum sampling methods. This novel approach provides a simple, accurate, low-cost tool for monitoring multidimensional anti-influenza hemagglutinin IgG responses in large population studies and clinical trials to comprehensively understand the effects of existing influenza virus antibodies on the immune response and new universal vaccine design.

The mPLEX-Flu assay can also be used to monitor the development of HA-specific antibodies against the influenza virus in infants. Tracking developing infant immunity to not only infection and vaccination strains but also other revolutionary strains would allow us to learn more about how OAS-type responses are first established. It would provide essential data for universal vaccine study and for understanding the mechanism of OAS.

### 6.2. Comprehensive Antigenic Study of HA Proteins

Right now, as discussed above, the broad neutralizing antibody (bnAb) activities still are the most important profiles of monoclonal antibodies to be considered. However, after the FluA-20 antibody was isolated, the broad cross-reactive antibodies (bcAbs) showed the protection against influenza virus that would likely be disregarded by traditional assays [51]. By contrast, MDA can detect non-neutralization activities of broad binding antibodies in a high throughput way. Absolutely, MDA will be a powerful serological assay for generating multidimensional data to exhibit the magnitude and breadth of binding to HAs from small amounts of sera.

### 6.3. Detecting Cross-Reactive Antibodies against Other Viral Proteins of Influenza Virus

Besides HA, neuraminidase (NA) also is an important target for inducing protective antibody- mediated responses [20]. Similarly, influenza virus M2 protein has an extracellular domain (M2e), which is highly conserved among influenza A viruses and B viruses. M2 is also being explored as a target for developing a “universal” vaccine to elicit the cross-protection against influenza. Unfortunately, little is known about the protective activity and broad cross-reaction of antibodies against these surface proteins.

At the same time, other internal proteins, such as nucleoprotein (NP) and the matrix protein 1 (M1), which are highly conserved between human seasonal and zoonotic influenza viruses [96], induce T-cell responses. These T-cell responses are shown to highly protect mice from the virus infection [97] and elicit robust CD8+ T cell responses across all human influenza A viruses [98]. After influenza virus infection, high circulating titers of NP Abs remain, and M1 antibodies can also be detected [96]. Currently, the effects of these antibodies against internal proteins on the T-cell response are unclear, especially the impact of pre-existing internal protein antibodies on the sequential humoral and cellular response.

Applying an expanded MDA panel that includes NA, NP and M1 protein-coupled beads in future influenza population surveys and clinical infection studies will allow us to evaluate antibodies against all these highly conserved proteins and HAs simultaneously. It would provide highly comprehensive data to help us to understand the T and B cell response to influenza virus infection, and also be beneficial for developing a “universal” influenza vaccine. While this review focuses on influenza, the underlying principles of MDA analysis apply equally to the study of immunity against other viruses that have multiple, antigenically similar strains.

## 7. Limitations of MDAs

As a novel technique, mPLEX-Flu and other MDAs also face some challenges and limitations. First, because MDAs detect the binding between the antibodies and HAs of influenza viruses, they are not able to directly reflect antibody protective function. By contrast, MN assays can estimate titers of neutralizing antibodies that inhibit influenza virus infection. However, our studies showed that data generated from mPLEX-Flu assay are highly correlated to HAI and MN data [31,63,64,76]. On the other side, HAI and MN assays have the chance to omit bcAbs that offer influenza virus protection through ADCC and ADCP, as discussed above. MDAs assay can be used to perform large scale screening, which can then be combined with other assays to identify and clarify the characters of antibodies.

Other limitations of MDAs are caused by the HA antigen coupled on the Luminex beads or microchips. First, most HA proteins used in the MDAs are expressed and purified by an insect cell baculovirus system that has to be glycosylated during post-translational modifications in infected insect cells. However, the insect cell glycosylation pathway is far simpler than those of human cells [99], even when both occur on the same N-glycosylation sites. Moreover, it has been reported that glycosylation of HAs is a crucial factor that needs to be considered when studying influenza infection and antigenic mutation (see review [100]). At present, it is not clear if differences in antigenic binding could be caused by the differences between insect cell-resourced HA proteins and those from human cells. Second, the density of HA proteins on the bead surface can vary, even when the coupling protocol and protein concentrations are kept consistent. This leads to error when comparing the binding between different influenza virus strains, which is similar to the error when comparing the HAI and MN titers between two viruses. To minimize this error, we established a unique method for generating standard curves for IgG binding to each virus strain [76].

## 8. Summary

The constantly changing HA antigenicity of influenza virus, along with the complexity of serological responses induced by the viruses in the human immune system, muddies efforts to interpret serology testing results. It had been commonly accepted that assessing the antibody response against only vaccine strain viral HAs is too restricted for understanding this complexity. Understanding the effects of pre-existing antibodies and cross-reactive antibodies against multiple strains’ HAs is becoming increasingly enticing in the influenza B cell immunity research field. MDAs and systems serology, the novel technologies combined with multidimensional data, computer modeling, and bioinformatics, are groundbreaking new tools for influenza vaccine study. They will open a novel comprehensive view to investigate the B cell response to influenza virus and be a powerful tool for universal vaccine development.

## Figures and Tables

**Table 1:** Multidimensional assay (MDA) methods for detecting antibodies against influenza HA strains.

Methods	Target Antigen	Species	Isotype	Sample Type(s)	Reference
Luminex array	NP, M1 and NS1 proteins	Chicken, turkey	IgY	Serum	[69]
	Whole HA of H1, H3, H5, Flu B	Human	IgA1, IgG1	Serum	[70]
	Whole HA of H1, H3, Flu B	Ferret, mouse, human	IgG, IgA, IgM	Serum	[67]
	Whole HA of H1, H3, H5	Human	IgG	Serum	[71]
	Whole HA of H1, H2, H3, H5, H7, H9, Flu B and chimeric HA	Human	IgG	Serum MBC culture	[63,72,73,74]
	Whole HA of H1, H2, H3, H5, H7, H9, Flu B	Human	IgG	Purified mAb	[31]
	Whole HA of H1, H2, H3, H5, H7, H9, Flu B and chimeric HA	Human	IgA, IgG	Breast milk Infant serum	[75]
	Whole HA of H1, H2, H3, H5, H7, H9, Flu B and chimeric HA	Human	IgG	Serum	[64,76]
	H1-16 whole HA, and N1-9 whole NA Avian flu	Chicken	IgY	Serum	[77]
Microarray	Random sequence peptides	Human	IgG	Serum	[65]
	Head domain of HA of H1, H2, H3, H5, H7, H9	Human	IgG	Serum or dry blood spots	[60,61,62,78,79,80,81]
	H1-H16 and H18 whole HA protein and/or HA peptides	Human	IgG	Serum	[66,82,83]
	H1-H18 whole HA	Chicken, duck, bat	IgY, IgG	Serum	[79,84,85]
Arrayed Imaging	H1, H3, H6, H5	Human	IgG	Serum	[86]
Reflectometry (AIR)	H1-H12 and Flu B	Mallard duck	IgY	Serum	[87]

## Abbreviations

The following abbreviations are used in this manuscript:

MDA	Multiple dimensional assay
mAb	monoclonal antibody
HA	hemagglutinin
NA	neuraminidase
MBC	memory B cell
HAI	hemagglutinin inhibition assay
MN	microneutralization assay
bcAb	broad cross-reactive antibody
bnAb	broad neutralizing antibody
